# Retention of the highly educated migrants: from the perspective of urban e-service capability

**DOI:** 10.1186/s12962-024-00509-4

**Published:** 2024-01-24

**Authors:** Fan Zhaoyuan, Liu Xiaofeng

**Affiliations:** 1 School of Information Management, Nanjing University, No. 163, Xianlin Avenue, Nanjing, 210093 China; 2 School of Sociology and Population Studies, Nanjing University of Posts and Telecommunications, No. 9, Wenyuan Road, Nanjing, 210023 China

**Keywords:** Urban e-service, New-generation highly educated migration, Settlement intention, China

## Abstract

**Background:**

Talent is a crucial resource for economic and social development, serving as the driving force behind urban progress. As China experiences rapid growth in digital city construction, the capability of e-services continues to improve incessantly. In China, the new-generation highly educated migrants (NGHEMs) account for ~ 20–30% of the total floating populations. This study aimed to explore the settlement intention of new-generation highly educated migrations in China from the new perspective of urban e-service capabilities. Furthermore, the mechanism of the urban e-services on the settlement intention on the NGHEMs will be proved.

**Methods:**

This paper employed data of China Migrants Dynamic Survey in 2017 and Evaluation Report of Government E-service Capability Index (2017). Descriptive analyses were conducted to investigate the factors influencing the settlement intention of NGHEMs in the destination city. Based on the principle of utility maximization, LASSO regression was employed to select individual and city characteristics that determined the settlement intention of NGHEMs. The impact of urban e-services on settlement intention was analyzed by using ordinal logit model. Additionally, robustness check, endogeneity analysis, and heterogeneity analysis were performed to validate the benchmark regression results. Finally, mediation model was employed to examine whether urban e-services enhance the settlement intention of NGHEMs by improving urban livability and urban innovation.

**Results:**

The results indicate that the urban e-services promote the NGHEMs’ settlement intention in the destination cities. Moreover, the results are still robust through a series of robustness tests. Furthermore, from the perspective of individual and regional heterogeneity, urban e-services significantly enhances the settlement intention of NGHEMs with male and female, married and urban household registration, and urban e-services can promote the settlement intention of NGHEMs with over 3 million inhabitants and those in the eastern regions of China. Finally, the intermediary effect test shows that urban e-services promote the settlement intention of NGHEMs through urban livability and urban innovation.

**Conclusion:**

This study highlights the important impact of urban e-services on the settlement intention of new-generation highly educated migrants. The conclusions of this study provide suggestions for the government to use when designing policies to enhance the settlement intention of the NGHEMs and to improve the development of urban e-services.

## Introduction

Knowledge accumulation and technological progress are the sources of sustained regional economic growth [1, 2]. As carriers of knowledge and technology, highly educated talents are the key drivers of national and regional development. The attraction and retention of talents have become a focus of international attention, as well as the more effective exploration of important talents [3]. The agglomeration of talents will produce various agglomeration and fusion effects, such as knowledge spillovers, low-cost communication and learning externalities, and specialized talent pool for industries that require high intelligence [4].

In recent years, China has undergone significant economic restructuring and industrial upgrading. In the “new normal” economy, technological innovation has become the core driving force for national and regional development [5, 6], talents are the primary resources driving technological innovation and regional economic and social development [7]. Chinese government attaches great importance to the talents and has formulated and implemented various talent policies and plans [8]. Chinese local governments attract talents through strategies for talent exploration, household registration system reform, improving public service capabilities, and building humanistic urban environment [9–12]. Recruiting and retaining talents is a comprehensive public policy, and the key to retaining talents lies in providing higher-level public services and creating a good urban environment. In recent years, China's local governments have formulated a series of public policies to retain highly educated talents [2, 13].

In addition, the migration is an inevitable phenomenon in the process of social and economic development [14].Since China’s economic reforms in 1978, the number and distance of migrants have continued to increase. According to the data from the 7th National Census of China, the migrant population in China has reached 376 million, and the migration structure has gradually become diversified, with the majority being highly educated migrants born after 1980, who are known as new-generation highly educated migrants (NGHEMs). According to the data from the 2015 nationwide 1% population sample survey, China has a population of 170.93 million with a university education or above, an increase of 42.87% compared to 2010. In the rapid process of economic development and urbanization, the new generation of highly educated migrants with higher competitiveness and higher quality human capital play an important role in optimizing the labor force age structure and alleviating the challenges of population aging in destination city. However, due to the social spatial mobility and residential instability of migrants, the floating of migrants is considered unsustainable for the future urbanization of China [15]. Therefore, the permanent settlement of NGHEMs in destination cities is crucial for sustainable urbanization in China.

Highly educated migrants may be attracted by the talent welfare policies of the destination city. The settlement intentions of these individuals are influenced by both the hard and soft power of the city, such as economy, environment, urban culture, urban governance, and high-level public service capabilities [16, 17]. Notably, the younger generation of Chinese individuals places significant emphasis on personal happiness [9], and the consideration of physical and mental health plays a vital role in determining their settlement intentions [18]. Therefore, cities that prioritize these aspects are more likely to attract and retain highly educated migrants. Under the background of the national strategy of vigorously promoting informatization, driving industrialization through informatization, promoting informatization through industrialization, and pursuing a new path of industrialization, urban e-services have emerged as a vital strategic initiative in China's informatization process [19, 20]. Amidst the “COVID-19” pandemic, the Chinese government effectively utilized digital technology and the urban e-service network to promptly disseminate information, with a result, the epidemic is controlled within a short timeframe successfully, and the production is restored gradually [21]. With the help of the digital technology and the urban e-service network, a more resilient society capable of withstanding risks is developed. In the digital era, urban digitization is the foundation of urban e-services, and the improvement of urban e-service capabilities can reciprocally facilitate the advancement of urban digitization. With the rapid development of digital city construction in China, the ability of digital public services improve unremittingly, it may take new impact mechanism on the long-term or permanent settlement intention of new-generation highly educated migrants (NGHEMs). However most research primarily focuses on the influence of household registration and public services [22, 23] on the settlement intention of China’s highly educated migrants. The research aims to address the following questions: How does the urban e-service capability impact the settlement intention of NGHEMs in China? What are the differences in the impact of urban e-services on the settlement intention of NGHEMs based on individual characteristics and the scale of the city? What mechanisms underlie the influence of the settlement intention of NGHEMs?

This paper is organized as follows: in “Literature review”, we review and summarize the literatures on the settlement intention of highly educated migrants, and propose theoretical hypotheses regarding the settlement intention of NGHEMs. In “Data and methodology”, we describe data, variable selection, and research methods of this paper. In “Results”, we measure the impact of urban e-service capabilities on the settlement of NGHEMs. We also test the robustness of the results, conduct heterogeneity tests, and investigate the mechanisms through a mediation model. Finally, in “Conclusion”, we conclude with the main findings and provide suggestions based on the results.

## Literature review

### Urban e-services and the settlement intention of highly educated migrants

In the past decades, there has been a flourishing research on internal migration in Western countries. The availability of early micro-census or survey data has enabled scholars to study the migration of specific groups. Many studies have focused on the migration and redistribution of educated or skilled labor force, who played a crucial role in economic growth during China's transition period. These studies include distribution patterns [24], migration modes [25], driving forces [26], spatial spillover effects [27], impact factors [28–30] and the impact of internal skilled labor migration on regional economies [7, 31]. Some studies indicate the existence of spatial agglomeration patterns of skilled talents [24, 26], and identify job opportunities as key factors [24]. The scale of interregional migration is positively related to the population stock in the origin and destination regions [32, 33].

With the development of China’s economy and urbanization, determinants of migrants' settlement intention are studied from different perspectives. One of these perspectives is urban public services. As a kind of public service, urban e-service has been studied more from the perspective of urban public services or social welfare to analyze the impact on the migrants' settlement intention. Some studies argue that talent migration is primarily driven by regional welfare differentials [34–36]. Some studies indicate that urban public service level is the key factor affecting the migrants' intention to stay, including educational public service, social citizenship public service, social insurance and urban public service quality [12, 37–41]. As highly educated migrants have higher social acceptance and more employment options, they face fewer barriers to migration. Highly educated migrants consider various factors including personal spatial preference, the quality of city services and governance, cultural adaptability, and opportunities for personal development when they choose destination cities to settle down [42–44]. So, they have higher expectations for the overall strength and attractiveness of the destination city. As urban industries and economies evolve, the demand for talent in cities grows steadily. Scholars have analyzed the public services on the determination of highly educated migrants’ settlement intention, such as high capacity of urban public services, advanced healthcare facilities, high-quality educational resources, high-quality smart city environment and well-established amenities, all of which make the destination city more attractive for highly educated immigrants to settle down [42–48]. Based on those theories, hypothesis 1 is proposed.

#### Hypothesis 1

For other factors being equal, urban e-service can promote the settlement intention of the new-generation highly educated migrants.

### Urban e-services, livability of city, and the settlement intention of new-generation highly educated migrants

E-services have requirements and characteristics of globality, strategic importance, public nature, hierarchy, ubiquity, and precision [49–52]. With the gradual expansion of national governance in China, encompassing both physical and digital realms and forming aggregated social networks, e-services place emphasis on “intelligent office,” “intelligent regulation,” “intelligent services,” and “intelligent decision-making” [53–55]. The impact of urban e-services on urban livability is reflected in the following two aspects, on one hand, e-services focus on prioritizing public needs, prioritizing service orientation, improving the efficiency of public services, providing efficient and convenient public services to the public, enhancing the transparency of administrative enforcement, promoting communication among the public, enterprises, and government, and reducing the costs of business and personal affairs [56–60]. On the other hand, e-services give full play to the value and role of data by adhering to the concept of “More data to run, less people to run”, it has greatly shortened the time needed for handling various business links, reduced the cost for enterprises and individuals to handle business, thus enhancing the livability of cities.

From the economic point of view, urban livability is usually considered as a kind of commodity, and people move their place of residence to meet the demand for this kind of commodity [61]. With the deepening of research, the connotation of urban livability is gradually enriched, it mainly includes elements such as natural conditions, public services, education, healthcare, transportation, and communication and cultural atmosphere [62–65].Enhancing urban livability is beneficial in meeting the growing demands of city residents for a better quality of life, thereby increasing the attractiveness of cities to migrants. Migrants choose to migrate to a particular city not only to secure higher salaries and employment opportunities but also to avail themselves of public services such as education and healthcare [66, 67]. The quality of urban public service provision has a significant positive impact on the settlement patterns of migrants [68, 69]. A higher level of urban livability contributes to improving residents' quality of life and fulfilling their aspirations for a better life. So, hypothesis 2 is proposed:

#### Hypothesis 2

Urban e-services enhance the settlement intention of new-generation highly educated migrants by improving urban livability.

### Urban e-services, urban innovation and the settlement intention of new-generation highly educated migrants

The impact of e-services on the level of regional innovation is mainly reflected in the following two aspects. On one hand, the implementation of e-services imposes higher demands on infrastructure, promoting technological upgrades and structural adjustments, and ultimately advancing the level of regional innovation directly [50, 54]. On the other hand, e-services can enhance technology management capabilities and optimize operational environments, thereby improving public service capabilities, optimizing service decision-making, and promoting resource sharing [52, 70]. This attracts the concentration of businesses and promotes entrepreneurial activities and innovation, ultimately raising the region's innovation level.

Under the background of entrepreneurship and innovation, the improvement of urban innovation and entrepreneurial levels can enhance the attractiveness of cities for migrants. Stable jobs and high incomes can improve the settlement intention of migrants [71, 72]. The labor force tends to choose jobs that can enhance their own productivity, as it helps them gain better employment prospects and higher salary returns. Based on the technological innovation driven by the urban e-services, highly educated migrants can work with higher efficiency and own higher economic income. Cities take the advantages of e-services to drive technological innovation and foster the clustering of upstream and downstream industries. This promotes the scale of economies, facilitates the spillover of knowledge and technology, and enhances the innovation capabilities and levels of enterprises and cities. The combination of innovation capabilities and income improvement will promote the settlement intention of highly educated migrants [71, 73]. Therefore, hypothesis 3 is proposed:

#### Hypothesis 3

Urban e-services enhances the settlement intention of new-generation highly educated migrants in destination cities by improving the urban innovation.

**Fig. 1:**
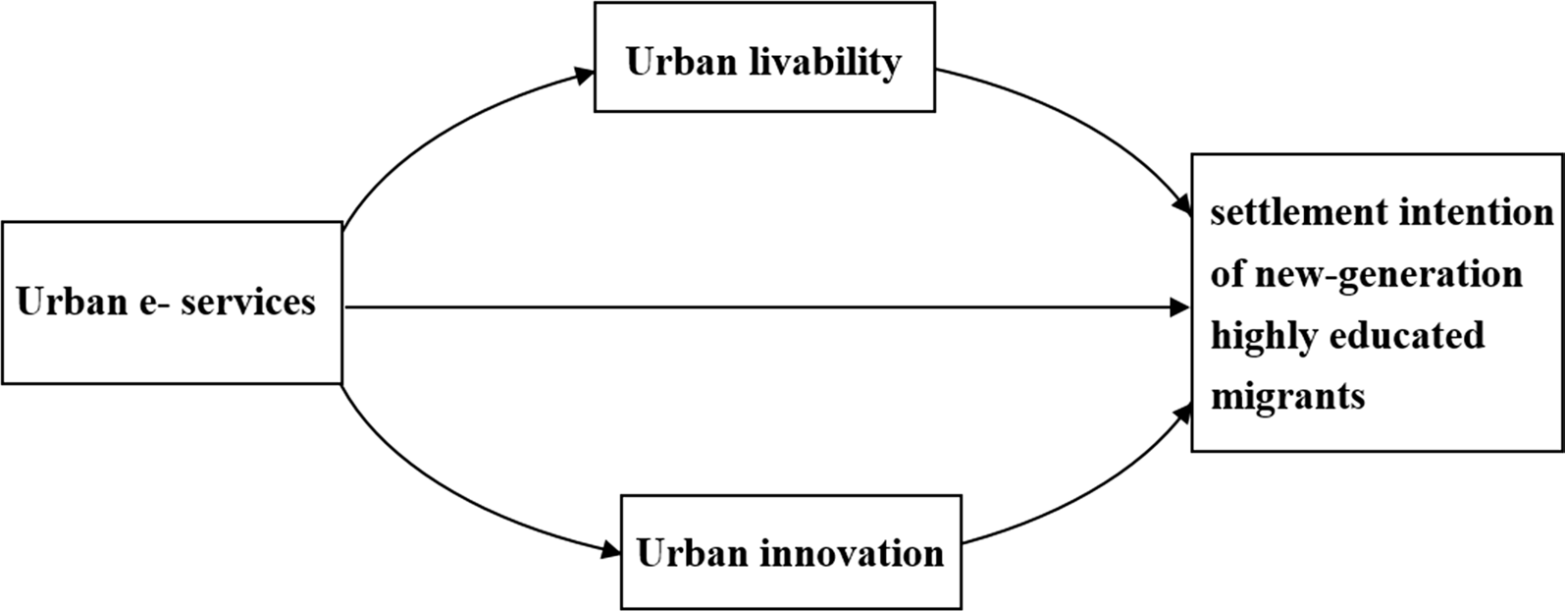
Theory frame of this work

Based on the review of the literature, the theory frame of this work is proposed, as shown in Fig. 1.

## Data and methodology

### Data source

The data utilized in this study was extracted from the China Migrants Dynamic Survey (CMDS) conducted in 2017 [74]. The CMDS is a nationally representative cross-sectional survey that provides open access to its data, the survey focused on migrants aged 16 and above who had relocated across county boundaries from their registered place of residence and had resided in their current location for at least one month. To ensure representativeness, the survey employed a stratified multi-stage random sampling method using the probability proportional to size (PPS) approach. A total of 169,989 samples from the floating population were collected across 348 cities, covering 32 provincial units in China. Additionally, data from the Evaluation Report of Government E-service Capability Index [75] were employed to assess urban e-service capability.

According to previous studies [42, 76, 77] and the widely acknowledged definition of talent in the current “talent wars” policies, this study focuses on the new-generation highly educated migrants who have migrated between cities and have not yet obtained local household registration in their destination city.” To ensure the credibility of the study, the data were meticulously chosen using the following criteria: (1) individuals with at least a college education; (2) individuals with different locations between household registration location and current residence cities; (3) individuals born between 1980 and 1999. After eliminating samples with significant missing information, a final dataset of 21,361 individual records was obtained by matching the data of migrants with urban e-services.

### Variable selection

#### Dependent variable

This paper constructs a comprehensive index system including 3 indicators: “urban stay intention”, “long-term city-residence intention” and “hukou transfer intention”. These indicators are used to assess the urban settlement intention of NGHEMs. The 3 indicators correspond to the following questions: “do you have plans to stay in this locality in the future”, “if you plan to stay, how long you expect to stay”, “if you meet the local settlement conditions, are you willing to transfer your household registration to the locality?”. Typically, migrants initially express the intention to stay in the city, which further leads to the desire for long-term settlement and hukou transfer.

In this paper, a value of 1 is assigned if respondents choose “consideration to stay in city” and “willing to transfer hukou into local city”, otherwise, the value is set to 0. For a stay duration of 6–10 years, a value of 1 is assigned, while a value of 2 is assigned for a stay duration exceeding 10 years, otherwise, the value is set to 0. The cumulative value of the 3 indicators, with equal weighting, represents the settlement intention of NGHEMs in destination cities. It is expressed using a scale of values (0, 1, 2, 3, 4), where 0 signifies “no settlement intention,” 1 denotes “low settlement intention,” 2 indicates “moderate settlement intention,” 3 represents “high settlement intention,” and 4 signifies “very high settlement intention.”

#### Core explanatory variable

E-service capability is the key variable being studied in this paper. The urban e-service level is measured based on multiple dimensions, including service provision capability, participation service capability, information service capability, and civil affair service capability provided by government microblogs, WeChat, and government apps, as outlined in the Evaluation Report of Government E-service Capability Index [75]. In this study, the composite index of WCI (WeChat Communication Index) and BCI (Micro-Blog Communication Index) is utilized to assess the urban e-service level, while the APP index is employed for robustness analysis.

#### Control variables

The variables that may impact the urban settlement intention of NGHEMs are categorized into individual-level variables and city-level variables. The individual-level variables encompass the following aspects: (a) individual characteristics variables, such as family members living together, gender, age, education level, hukou (household registration), party membership, and marital status; (b) economic characteristics, including total monthly household income and total monthly housing expenses over the past year, as well as whether the NGHEMs possesses self-owned housing; (c) migration characteristics variables, such as the total number of cities migrated to, whether the father or mother has had migration experience, migration alone, the level of interaction with local residents during leisure time, the extent of geographical floating, and the duration of migration; (d) health variables, including self-perceived health condition, whether they have established a health record in the past year, and whether they have experienced any illnesses or health issues in the previous year; (e) social integration variables, such as whether they pay attention to the changes in the city they currently reside in, their willingness to assimilate with the local community, and whether they have rural cooperative medical insurance or a personal social security card. The city dimensional variables include per capita GDP, number of hospital beds per thousand, and the number of primary and secondary school teachers per thousand. The data for these variables were obtained from the China City Statistical Yearbook [78]. These control variables have been selected for inclusion in this study.

#### Mediating variables

Urban livability: Urban livability is assessed based on five indicators: (1) education level, measured by the student-to-teacher ratio in regular higher education institutions, in regular vocational schools, in regular secondary schools, and in regular primary schools; (2) healthcare facilities, including number of hospitals and clinics per 10,000 people, number of hospital beds per 10,000 people, number of licensed or assistant doctors per 10,000 people; (3) public transportation, evaluated by the number of taxis per 10,000 people and the number of public buses and trams per 10,000 people; (4) environmental quality, gauged by the comprehensive utilization rate of general industrial solid waste, the centralized treatment rate of sewage treatment plants, the harmless treatment rate of household waste, and the extent of green coverage in urban areas; (5) online living, measured by the ratio of internet broadband access users and mobile phone users [79]. All indicators are positive attributes, and the entropy weighting method [80] is utilized for the analysis and calculation of urban livability indicators. The data is sourced from the 2017 “China Urban Statistical Yearbook.” [78].

Urban innovation: Urban innovation is determined by the city innovation index derived from the “China's city and industry innovativeness report 2017 “[81]. A higher innovation index signifies a stronger innovation capability of the city.

### Model

In this study, descriptive analysis of variables was initially conducted using Stata 15.1. The Lasso model was then utilized for variable selection. Subsequently, the logistic regression analysis was employed to examine the precise impact of urban e-service on the settlement intention of NGHEMs. Finally, a mediation model was employed to analyze the potential influence mechanisms.

#### Lasso model

To address the issue of multicollinearity in the data and mitigate potential estimation bias of the variables, the maximization process of the likelihood function with a Lasso penalty term is employed. This allows for the estimation of Lasso regression coefficient values and selects the relevant variables. The model can be expressed as:

1 $$\overset{\lower0.5em\hbox{$\smash{\scriptscriptstyle\frown}$}}{\beta } { = }\arg \min \left\| {y - \sum\limits_{j = 1}^{p} {\left( {\beta eservice_{ij} + \theta X_{ij} + \alpha city_{ij} } \right)} } \right\|^{2} + \lambda \sum\limits_{j = 1}^{P} {\left| {\beta_{ij} } \right|} ,$$

where $\lambda$ is the nonnegative canonical adjustment coefficient and $j$ is the number of variables. Lasso method will set the coefficients value of unimportant variables as 0. By utilizing the Sklearn Library in Python, the LassoCV function is employed to eliminate variables with coefficient equal to 0, so that the unimportant variables can be deleted. The Lasso penalty term penalizes each regression coefficient, resulting in an estimator deviation. Therefore, an ordinal logit regression is constructed for the variables selected, the maximum likelihood estimation method is employed to obtain consistent estimates of regression coefficients and statistically significant results, effectively mitigating estimation bias in limited samples, as a result, the variables are delimited, including party membership, rent, whether they have more interaction with local residents, whether they have experienced any illnesses or health issues in the previous year, whether they have participated in the free medical care program.

#### Ordinal logit model

The settlement intention of NGHEMs in destination cities is presented by a value (0, 1, 2, 3, 4), with a higher value indicating a stronger settlement intention. Therefore, the probability of urban settlement intention for NGHEMs can be expressed as:

2 $$\log\,(p_{ij} ) = \ln \left( {\frac{{p_{ij} }}{{1 - p_{ij} }}} \right) = \alpha + \beta eservice_{ij} + \theta X_{ij} + \alpha city_{ij} ,$$

where $p_{ij} = P\,(y \le j|x)$ denotes the cumulative probability that settlement intention of NGHEMs takes preceding j values. $p_{ij}$ denotes the response probability for individual i in city j.$\alpha$ is a constant, $eservice$ is the urban e-service. $X$ is micro variables, $city$ is the county-level variables; $\beta$ and $\theta$ are the estimated coefficients of independent variables.

#### Mediating effect model

Theoretical analysis indicates that the capability of urban e-services can influence the settlement intention of NGHEMs by altering urban livability and employment rate. To validate it, we establish the following three-step mediation model for verification:

3 $$y_{ij} = \alpha + \beta eservice_{ij} + \delta X_{ij} + \nu city_{ij} + \varepsilon_{i} { ,}$$

4 $$MID_{ij} = \alpha + \gamma DIG_{ij} + \varphi X_{ij} + \sigma city_{ij} + \varepsilon_{i} {,}$$

5 $$y_{ij} = \alpha + \phi eservice_{ij} + \theta MID_{i} + \varphi X_{i} + \eta city_{ij} + \varepsilon_{i} ,$$

where, $y_{ij}$ represents the settlement intention of NGHEMs, $eservice_{ij}$ represents the capability of urban e-services, $MID_{i}$ represents urban livability or urban innovation,$X_{i}$ is control variable,$\alpha ,\;\beta ,\;\delta ,\;\gamma ,\;\phi ,\;\theta$ are the coefficients of variables, respectively, $\varepsilon_{i}$ is error term.

The verification process is as follows: (1) Test the significance of the coefficient $\beta$. If it is significant, proceed to step (2); otherwise, there is no mediating effect. (2) Perform consecutive tests on $\gamma$ and $\theta$, if both coefficients are significant, continue with the test for $\phi$_._ If $\phi$ is significant, it indicates a significant mediating effect; otherwise, there is a complete mediating effect. If either $\gamma$ or $\theta$ is not significant, conduct a Sobel test. The statistical value is calculated as $Z = \gamma \theta /(\gamma^{2} s_{\gamma }^{2} + \theta^{2} s_{\theta }^{2} )$, where $s_{\gamma }^{2}$ and $s_{\theta }^{2}$ are the the standard deviation of $\gamma$ and $\theta$, respectively. If Z is significant, it indicates the presence of a mediating effect, and the magnitude of the mediating effect can be calculated using $\gamma \theta {/(}\gamma \theta { + }\beta_{{2}} {)}$, otherwise, there is no significant mediating effect.

## Results

### Descriptive analysis

According to Table 1, the settlement intention of NGHEMs is between “medium settlement intention” and “high settlement intention”. The average urban e-service capability is 0.549. Regarding individual characteristics variables, the majority of the respondents were rural residents, married, and non-party members and league members, and with an average of 2.7 family members living together. Additionally, the logarithm of the total monthly household income for NGHEMs is 8.941, equivalent to an income of approximately 8000 yuan, and majority of them do not own a house. In terms of migration characteristics and health status, the average number of cities where NGHEMs have floated is 1.837, indicating that most have migrated to one or two cities. The majority of respondents have migrated alone, have limited interaction with local registered residents, migrated inner-provincial, possess no health records, have poor health conditions, have been migrating for about 4–5 years, and have parents without migrant experience, ~ 31.6% of respondents have participated in rural cooperative medical insurance, while about 72.5% possess personal social security cards. They care the change happening in the city they live in and express a desire to blend in with the local population. As for the city-level variables, number of primary and secondary school teachers per 1,000 population is 8.722, and the number of hospital beds per 1000 inhabitants is 7.111. The sample characteristics align closely with the data from China’s Seventh National Population Census, indicating the credibility and representativeness of the survey samples and data.

**Table 1:** Variable selection and basic description

Variable	Implication	Classification	Mean	Standard deviation	Min.	Max.
S-intention	Settlement intention of NGHEMs		2.587	1.410	0	4
e-services	Urban e-service capability	Core variable	0.549	0.174	0.027	0.824
FamilyNum	Number of family members living together	Individual characteristics	2.713	1.172	1	10
Gender	Female = 0, male = 1		0.459	0.498	0	1
Age	Age		28.990	4.142	17	37
Education	Educational attainment: associate’s degree = 5, bachelor’s degree = 6, postgraduate degree = 7		5.440	0.557	5	7
Hukou	Agricultural hukou = 0, non-agricultural hukou = 1		0.469	0.499	0	1
Political status	Party members and league members = 1, others = 0		0.328	0.469	0	1
Marriage	Married = 1, others = 0		0.694	0.461	0	1
ln(Income)	Logarithm of total monthly household income	Economic characteristics	8.941	0.749	0	12.206
ln(Houseexp)	Logarithm of total monthly housing expenditure		5.208	3.327	0	10.309
House	Self-owned house = 1, others = 0		0.387	0.487	0	1
Totalcity	Total number of floating cities	Migration characteristics	1.837	1.210	1	40
ParentE	Father or mother with floating experience = 1, others = 0		0.294	0.456	0	1
MigAlone	Migration alone = 1, others = 0		0.712	0.453	0	1
InterLocal	Intersection more with local registered residence = 1, other = 0		0.493	0.500	0	1
MigScope	Regional migration: interprovincial = 1, and inner-provincial = 0		0.450	0.498	0	1
DurTime	Years duration in host city		4.546	4.214	0	35
Health	Health condition, the higher the value, the better the health	Health status	1.188	0.582	1	4
HealthRec	Establishment of health record = 1, otherwise it is 0		0.287	0.452	0	1
HealthNow	In the last year, suffering illness or indisposed = 1, others = 0		0.534	0.499	0	1
Carecity	Paying more attention to the change of the city lived, the greater the value	Social integration	3.434	0.572	1	3
Locallife	Wiliness to blend in with the locals		3.442	0.581	1	3
Rural-med	Participating in rural cooperative medical insurance = 1, others = 0		0.316	0.465	0	1
Soc-card	Personal social security card = 1, others = 0		0.725	0.447	0	1
Pergdp	Logarithm of GDP per capita	City level	11.293	0.461	9.384	12.281
Teachernum	Number of primary and secondary school teachers per 1000		8.722	2.779	3.376	25.852
Bednum	Number of hospital beds per 1000		7.111	1.950	1.431	13.863

### Benchmark regression

Table 2 presents the regression results of urban e-service capability’s influence on the urban settlement intention of NGHDMs, in which the ordinal logit model is used. In model 1, the influence of urban e-service capability on the settlement intention of NGHDMs is examined. The regression coefficient of urban e-services is 1.430, which is statistically significant at the 5% level. The regression results indicate that the enhancement of urban e-services contributes to an increased likelihood of NGHEMs choosing to reside in the city. Specifically, a 1% improvement in urban e-services corresponds to a 1.43% increase in the probability of NGHDMs selecting the city as their settlement destination. In model 2, individual characteristics variables of NGHDMs are considered in addition to model 1. Model 3 incorporates economic characteristics variables of NGHDMs based on model 2. Model 4 incorporates migration characteristics variables of NGHDMs based on model 3. Model 5 incorporates health condition variables of NGHDMs based on model 4. Model 6 incorporates social integration variables of NGHDMs based on model 5. Finally, model 7 includes city-level variables based on model 6. The regression coefficient for urban e-services remains significantly positive across all models. The regression results from all models consistently demonstrate that a higher urban e-service capability has a positive impact on the settlement intention of NGHDMs in destination cities, confirming hypothesis 1.

**Table 2:** Benchmark regression results

variable	Model 1	Model 2	Model 3	Model 4	Model 5	Model 6	Model 7
e-services	1.430** (0.563)	1.221*** (0.435)	1.190*** (0.408)	1.052*** (0.356)	1.103*** (0.423)	0.983*** (0.327)	0.714** (0.281)
FamilyNum		0.129*** (0.028)	0.022 (0.020)	− 0.014 (0.019)	− 0.014 (0.019)	− 0.006 (0.019)	0.016 (0.018)
Gender		− 0.273*** (0.041)	− 0.251*** (0.039)	− 0.250*** (0.039)	− 0.244*** (0.040)	− 0.221*** (0.039)	− 0.209*** (0.038)
Age		0.043*** (0.008)	0.034*** (0.007)	0.020*** (0.005)	0.021*** (0.006)	0.018*** (0.006)	0.019*** (0.005)
Education		0.225*** (0.049)	0.115*** (0.034)	0.119*** (0.034)	0.119*** (0.034)	0.075** (0.035)	0.070** (0.033)
Hukou		0.485*** (0.046)	0.403*** (0.044)	0.401*** (0.044)	0.398*** (0.044)	0.274*** (0.052)	0.290*** (0.047)
Marriage		0.730*** (0.060)	0.465*** (0.063)	0.569*** (0.062)	0.559*** (0.060)	0.550*** (0.061)	0.540*** (0.056)
ln(income)			0.328*** (0.051)	0.310*** (0.042)	0.314*** (0.043)	0.290*** (0.043)	0.228*** (0.039)
House			0.902*** (0.100)	0.835*** (0.103)	0.835*** (0.102)	0.753*** (0.102)	0.760*** (0.098)
Totalcity				− 0.036*** (0.012)	− 0.033*** (0.011)	− 0.032** (0.013)	− 0.020 (0.014)
ParentE				0.077** (0.036)	0.085** (0.036)	0.083** (0.037)	0.079** (0.035)
MigAlone				0.187*** (0.033)	0.188*** (0.033)	0.158*** (0.033)	0.159*** (0.033)
MigScope				0.029 (0.26)	0.037 (0.110)	0.073 (0.104)	0.010 (0.091)
DurTime				0.055*** (0.007)	0.054*** (0.007)	0.048*** (0.007)	0.047*** (0.006)
Health					− 0.127*** (0.035)	− 0.077** (0.031)	− 0.075*** (0.039)
HealthRec					0.199*** (0.045)	0.117** (0.045)	0.140*** (0.039)
Carecity						0.155*** (0.033)	0.155*** (0.034)
locallife						0.801*** (0.040)	0.808*** (0.038)
Rural-med						− 0.241*** (0.051)	− 0.224*** (0.047)
Soc-card						0.100* (0.057)	0.116** (0.053)
Pergdp							0.274** (0.134)
Teachernum							0.081** (0.036)
Bednum							− 0.045*** (0.016)

“***”, “**”, “*” are significant at 1%, 5%, and 10% significant levels respectively, the standard errors clustered by city are in parentheses, the same below

Model 7 includes all control variables, so we will primarily discuss the results from this model. The individual characteristics variables of gender, age, education level, household registration, and marital status have statistically significant positive effects on the urban settlement intention of NGHEMs. Gender has a significant negative impact on the urban settlement intention of NGHEMs, compared to females, males are less likely to choose the migrant cities as their settlement destination. The settlement intention of NGHEMs is higher among those with characteristics such as older age, higher education level, urban household registration, and being married, which is consistent with the research findings of Zhu Huizhen et al. [82].

For economic characteristics variables, higher income and owning housing in city have a positive impact on the settlement intention of NGHEMs in destination cities [83–85]. For health variables, having an urban health record and experiencing poorer physical health are factors that promote the settlement intention of NGHEMs, possibly due to the better medical care and social security systems available in cities. For migration characteristics variables, migrants who have parents with migration experience, longer migration history, have migrated alone, and have interprovincial migration, will facilitate the settlement intention of NGHEMs in destination cities, which aligns with the findings of a study conducted by Yang and Fan in 2019 [86]. As Longer duration in destination cities allows NGHEMs to establish more relationships and have access to more job opportunities [87], thereby facilitating their integration into urban society and strengthening their settlement intention. In terms of social integration variables, regression results indicate that NGHEMs who are interested in the city's dynamics, willing to integrate into local communities, and have applied for urban social security cards exhibit a higher settlement intention in destination cities. For city-level variables, higher per capita GDP and a greater number of hospital beds per thousand people are associated with a higher settlement intention of NGHEMs. However, having more primary and secondary school teachers per thousand people reduces the settlement intention of NGHEMs. This can be attributed to the inefficient allocation of teacher resources in small and medium-sized cities, where the teacher resources ratio tends to be lower compared to larger cities. As small and medium-sized cities experience population outflows, the smaller number of teachers per thousand people negatively affects the settlement intention of NGHEMs in destination cities.

### Robustness analysis

The robustness of the regression results is verified from three aspects: (1) ordinal probit regression and poisson regression, (2) instrumental variable method, and (3) regression with intercepted data. The regression results are shown in Table 3.

1. Ordinal probit regression and poisson regression. Model 8 and model 9 in Table 3 give the regression results of ordinal probit regression and poisson regression respectively, and the results are consistent with the previous results.
2. Instrumental variable method. There may be a correlation between e-services capability and the settlement intention of NGHEMs in destination cities. To address endogeneity issues, the instrumental variable method was utilized. The capability of government APP services was employed as an instrumental variable for robustness testing. Correlation analysis revealed a strong positive correlation (correlation coefficient = 0.308, significant at the 1% level) between the capability of government APP services and the “Dual Micro” electronic services. To ensure the validity of the instrumental variable, a weak instrument test utilizing the Kleibergen-Paap Wald F statistic was conducted [88]. The results show that the value of the F statistic was significantly greater than 10, indicating the absence of weak instrument problems. Therefore, the selection of the capability of government APP services as the instrumental variable was deemed valid. Both LIML and 2SLS estimations yielded consistent results. The regression results of Model 10, estimated by LIML, provided strong evidence that urban e-services promote the settlement intention of NGHEMs.
3. Regression with intercepted data. To validate the robustness of the regression results, in model 11, intercepted data was used due to potential variations in the multivariate data. The first 1% and the last 1% of the urban e-service level variables were eliminated. The results confirmed the robustness of the previous findings.

**Table 3:** Robustness analysis

Variable	Model 8	Model 9	Model 10	Model 11
e-service	0.446*** (0.167)	0.144*** (0.023)	1.454*** (0.253)	0.686*** (0.282)
Control variables	Yes	Yes	Yes	Yes
Pseudo R^2^	0.105	0.060	0.278	0.104
Quantity of samples	21,361	21,361	15,425	20,873

*** is significant at 1% significant levels, the standard errors clustered by city are in parentheses

The results of the robustness tests show that China's urban e-service capability promotes the settlement intention of NGHEMs in destination cities.

### Heterogeneity analysis

Based on the assumption that the migrants have the same preference for government public services, this part analyzes the settlement intention of NGHEMs from multiple perspective. To examine the heterogeneity effect of e-service capability on the settlement intention of NGHEMs, the ordinal logit regression model was employed.

### Gender, marriage and hukou (household registration)

Table 4 shows the regression results of the urban e-services on the settlement intention of NGHEMs, stratified by gender, marital status, and household registration. The regression results of model 12 and model 13 reveal that urban e-services can significantly promote the settlement intention of both male and female NGHEMs. For each unit of improvement in e-service level, the probability of NGHDMs selecting the city as their settlement destination increased by 0.784 units, while for females it increased by 0.682 units.

**Table 4:** Heterogeneity analysis—gender, marriage and registered residence

variable	Model 12 (male)	Model 13 (female)	Model 14 (married)	Model 15 (unmarried)	Model 16 (rural)	Model 17 (city)
e-services	0.784*** (0.224)	0.682* (0.361)	0.924*** (0.310)	0.318 (0.291)	0.417 (0.281)	1.117*** (0.332)
Control variables	Yes	Yes	Yes	Yes	Yes	Yes
Pseudo R^2^	0.100	0.113	0.091	0.073	0.093	0.104
Quantity of samples	9806	11,555	14,821	6541	11,352	10,010

***, * are significant at 1%, and 10% significant levels respectively, the standard errors clustered by city are in parentheses

Model 14 and model 15 examine the influence of marital status on the settlement intention of NGHEMs. The regression results demonstrate that urban e-services have a positive impact on the settlement intention of married NGHEMs, but has little effect on single NGHEMs. This could be attributed to the lower migration costs for single NGHEMs, making them less sensitive to urban e-service capability.

Models 16 and 17 consider urban and rural household registration. The findings reveal that urban e-services positively affect the settlement intention of NGHEMs with both urban and rural household registration, with a stronger effect observed among those with urban household registration. For each unit improvement in e-service level, the probability of NGHDMs selecting the city as their settlement destination with urban household registration increased by 1.117 units.

To attract a larger number of settlers, cities should prioritize the improvement of urban e-service capability along with China's urbanization rate.

### City size and regionalization

In 2019, the General Office of the Communist Party of China Central Committee and the General Office of the State Council have issued the “Opinions on Promoting the Reform of the System and Mechanism for the Social Mobility of Labor and Talent”. According to the opinions, the settlement restrictions were canceled for cities with a population of less than 3millions.

Table 5 shows the regression results of the urban e-services on the settlement intention of NGHEMs, stratified by city size and regionalization. In model 18 and model 19, the settlement intentions of NGHEMs in destination cities is examined. Model 18 focused on cities with a permanent resident population in the city exceeding 3 million, while Model 19 focused on cities with a population less than 3 million. The regression results show that urban e-services have a significant impact on the settlement intention of NGHEMs in cities with a population exceeding 3 million. For each unit of improvement in e-service capability, the probability of NGHDMs selecting the city as their settlement destination will increase by 0.709 units. In contrast, for cities with a population less than 3 million, with a 0.415 unit increase in settlement intention for each unit improvement in e-service capability. The results imply that NGHEMs in big cities are more sensitive to e-service capability.

**Table 5:** Heterogeneity analysis—city size and regionalization

Variable	Model 18 (more than 3million)	Model 19 (less than 3milloin)	Model 20 (east)	Model 21 (central)	Model 22 (west)
e-services	0.709** (0.362)	0.415* (0.218)	1.035** (0.424)	0.149 (0.367)	0.811* (0.439)
Control variables	Yes	Yes	Yes	Yes	Yes
Pseudo R^2^	0.129	0.080	0.135	0.081	0.070
Quantity of samples	12,643	8718	11,706	4599	5056

**, * are significant at 5%, and 10% significant levels respectively, the standard errors clustered by city are in parentheses

Model 20–22, respectively examines the impact of urban e-service capability on the settlement intention of NGHEMs in the eastern, central and western regions of China. The results indicate that the impact of e-service capability varies across regions, with the eastern region having the highest impact, followed by the western region, and finally the central region. In the eastern region, each unit improvement in e-service capability leads to a 1.035 unit increase in the probability of NGHDMs selecting the city as their settlement destination. In the western region, this impact is slightly lower at 0.811 units. The results suggest that the settlement intentions of NGHEMs may be influenced by the regional differences in China, with the NGHEMs in the eastern region showing greater sensitivity to e-service capability.

### Mechanism analysis

The above analysis show that urban e-services play a crucial role in increasing the settlement intention of NGHEMs in destination cities. To further validate the impact mechanisms, we utilizes mediating effect model to investigate.

*Urban livability*: Building upon the previous analysis, urban e-services can enhance the settlement intention of NGHEMs by improving the livability of cities. To verify hypothesis 2, a mediating effect model was employed to investigate the mechanism that urban livability influences the settlement intention of NGHEMs. The results are shown in Table 6 (1)–(3). The results in Table 6 (1) demonstrate that urban e-services have a significant positive impact on the settlement intention of NGHEMs, with a significance level of 5%, which indicates that urban e-services promote the settlement intention of NGHEMs in destination cities. In Table 6 (2), the positive regression coefficient of urban e-services on urban livability indicates that urban e-services contribute to the improvement of cities' overall livability. The results in Table 6 (3) reveal a significantly positive regression coefficient for both urban e-services and urban livability on the settlement intentions of NGHEMs. This result indicates the presence of a mediating effect, whereby urban livability serves as an intermediary between urban e-services and the settlement intentions of NGHEMs. In other words, urban e-services have a positive impact on the settlement intentions of NGHEMs by creating a more livable urban environment.

**Table 6:** Mechanism validation

Variable	S-intention (1)	urban livability (2)	S-intention (3)	S-intention (4)	Innovation (5)	S-intention (6)
e-services	0.602** (0.275)	0.023*** (0.006)	15.183*** (1.725)	0.687** (0.281)	54.502** (22.940)	0.286*** (0.098)
Urban livability			0.251** (0.124)			
Urban innovation						0.008*** (0.0008)
Control variables	Yes	Yes	Yes	Yes	Yes	Yes
R^2^	0.084	0.874	0.086	0.107	0.571	0.109
Quantity of samples	13,227	13,227	13,227	20,832	20,832	20,832

***, ** are significant at 1%, and 5% significant levels respectively, the standard errors clustered by city are in parentheses

*Urban innovation*: Innovation serves as a powerful driving force for the high-quality development of both cities and enterprises, as the urban innovation capacity directly influences the economic development and overall quality of the city. Through the promotion of technological innovation in enterprises, employee welfare can be enhanced and more job opportunities can be created, thereby facilitating the retention of population. To verify hypothesis 3, a mediating effect model is employed to investigate the mechanism that urban innovation influences the settlement intention of NGHEMs.

The results are shown in Table 6 (4)–(6). The results in Table 6 (4) show that urban e-services have a significantly positive impact on the settlement intention of NGHEMs, with a significance level of 5%, which indicates that urban e-services promote the settlement intention of NGHEMs. In Table 6 (5), the positive regression coefficient of urban e-services on urban innovation indicates that urban e-services contribute to the improvement of urban innovation capacity. The results in Table 6 (6) reveals a significantly positive regression coefficient for both urban e-services and urban innovation on the settlement intention of NGHEMs. This result indicates the presence of a mediating effect, whereby urban innovation serves as an intermediary between urban e-services and the settlement intentions of NGHEMs. In other words, urban e-services have a positive impact on the settlement intentions of NGHEMs by leveraging the innovation capacity of cities.

## Limitations

Cities, as the drivers of new urbanization, play a vital role in attracting and retaining the new-generation highly educated migrants, thereby supporting China's economic and social development. These new-generation highly educated migrants are highly floating, making it crucial to understand how to attract and retain the talents in order to promote urban economic growth. This paper focuses on the influence of urban e-service capability on the settlement intention of NGHEMs in destination cities. The influence mechanisms are analyzed from the individual characteristics, economic characteristics, migration characteristics, health characteristics, and social integration characteristics, and city-level characteristics. Furthermore, it is recommended that additional factors with potential influence on settlement intentions, such as psychological pressure, house price levels, and the quality of living environments, should be considered for further study.

Additionally, the uneven distribution of resources leads to imbalances in economic development among regions, with economically advanced areas like Beijing, Tianjin, Jiangsu, Zhejiang, and Shanghai being favored destinations for NGHEMs. The concentration of talents is particularly prominent in eastern China. In the context of the new competition for talents, it is important to examine and explore the impact of preferential policies implemented in different regions.

## Conclusion

This paper utilizes China Migrants Dynamic Survey in 2017, Evaluation Report of Government E-service Capability Index (2017), and the China Statistical Yearbook data of 2017. An ordinal logit model is employed to examine the impact of urban e-service capability on the settlement intentions of new-generation highly educated migrants in destination cities. The results reveal that urban e-services have a significant and positive influence on the settlement intention of NGHEMs. Through a series of robustness tests, the results remain robust and consistent.

Furthermore, the analysis of individual and regional heterogeneity indicates that urban e-services notably enhance the settlement intentions of both male and female NGHEMs, as well as those who are married and possess urban household registration. Moreover, the positive effect of urban e-services are more pronounced among NGHEMs in cities with a population above 3 million and those in the eastern part of China. Subsequent tests suggest that this influence is mediated by the enhancement of urban livability and urban innovation.

Consequently, it is recommended that the government prioritize the improvement of e-service capability in Chinese cities, particularly in larger cities and the eastern region. Simultaneously, attention should be given to enhancing urban livability and urban innovation, as these factors play a crucial role in influencing the settlement intentions of NGHEMs. Although the data employed in this study are specific to China, the findings can potentially serve as a theoretical basis for enhancing population migration policies globally.

## Data Availability

The datasets generated and/or analyzed during the current study are available here: https://www.chinaldrk.org.cn/wjw/#/data/classify/population/yearList.
